# Horner syndrome as a complication after thyroid microwave ablation: Case report and brief literature review

**DOI:** 10.1097/MD.0000000000011884

**Published:** 2018-08-24

**Authors:** Xi Zhang, Yunhao Ge, Peiyou Ren, Jia Liu, Guang Chen

**Affiliations:** a Department of Thyroid Surgery, First Hospital of Jilin University; b Department of Neurology, People's Hospital of Changchun City, Jilin, China.

**Keywords:** case study, cervical sympathetic trunk, Horner syndrome, oculosympathetic pathway, review, thyroid microwave ablation, thyroid radiofrequency ablation

## Abstract

**Rationale:**

Horner's syndrome (HS) can present as a complication of thyroid surgery, particularly after thyroid microwave ablation (MWA). HS presents clinically with eyelid ptosis, miosis, enophthalmos, anhidrosis, and vascular dilatation, all of which result from a damaged oculosympathetic chain. We aimed to try to avoid such devastating symptoms in future cases by exploring reasons for the destruction of the cervical sympathetic trunk.

**Patient concerns:**

HS has previously been reported in the literature as a complication of thyroid surgery. Here, we report the case of a 44-year-old female patient who presented with miosis and eyelid ptosis following thyroid MWA.

**Diagnoses:**

This patient was subsequently diagnosed with HS.

**Interventions:**

Mecobalamin was administered immediately.

**Outcomes:**

After 5 months of follow up, the patient's miosis and ptosis was incompletely relieved.

**Lessons:**

Although HS is a rare complication of thyroid MWA, surgeons must be aware of the anatomic relationship of the cervical sympathetic trunk and thyroid gland with adjacent structures. Moreover, we hope this case presentation enables surgeons to take measures to minimize the possibility of oculosympathetic damage. Long-term follow up and comprehensive assessments are important for the patient's prognosis.

## 1 Introduction

Horner syndrome (HS) presents clinically with the following symptoms: eyelid ptosis, miosis, enophthalmos, anhidrosis, and vascular dilatation. These symptoms result from a damaged oculosympathetic chain and the condition was first described by Swiss ophthalmologist Johann Friedrich Horner in 1869.^[1]^ As a rare complication of thyroid surgery, HS has only been reported in a handful of papers and no previous papers have reported the condition following thyroid microwave ablation (MWA).

Ultrasound-guided thyroid MWA has been adopted worldwide as a relatively novel technology. Thermal ablation, including microwave, radiofrequency, and laser ablation, is a focus of research for the treatment of various space-occupying lesions. It is conducted with the guidance of ultrasound and is mainly used to treat liver, lung, kidney, and breast cancer. As an innovative alternative to surgical treatment for benign thyroid nodules, thermal ablation can effectively reduce the volume of nodules. This technique can cause coagulative necrosis of the lesion tissue via high temperature heating, enabling minimally invasive local inactivation of the lesion.^[2,3]^ The essence of MWA is the electromagnetic wave. The magnetron in the microwave generator produces microwaves, which can raise the local temperature to 60 to 100 °C and leads to protein denaturation.^[4]^ In 2000 and 2001, Pacella^[5]^ and Dupuy^[6]^ first applied laser ablation and radiofrequency ablation (RFA), respectively, to treat recurrent thyroid carcinoma. Currently, RFA and MWA are mainly used to treat benign thyroid nodules in China.

As of today, thyroid MWA has been administered to 250 patients in our department, and among these we observed 1 case of HS in a female patient. A few previous cases of HS after conventional thyroid surgery or video-assisted thyroidectomy have been reported, but this is the first reported case of HS after thyroid MWA.

The present study was approved by the institutional ethics committee of the First Hospital of Jilin University. The patient provided consent for the case to be published.

## 2 Case presentation

**Figure 1:**
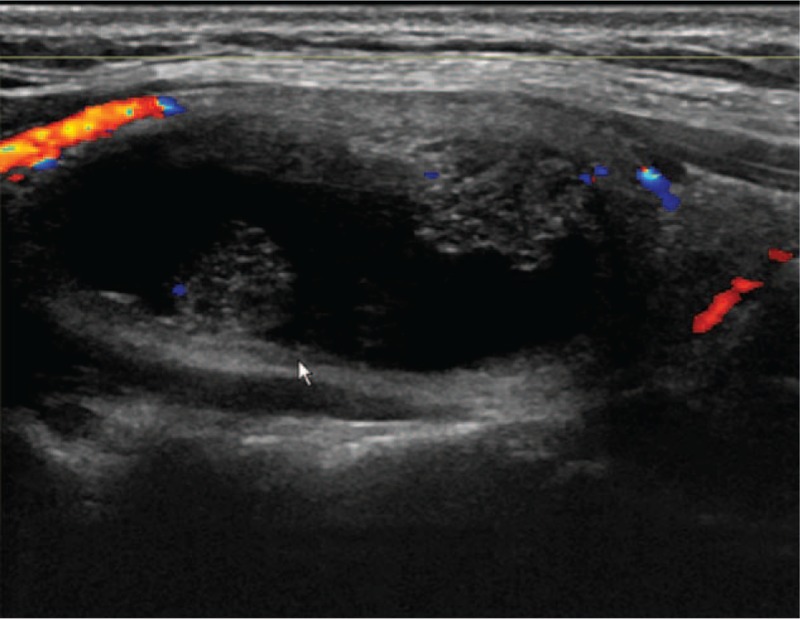
Longitudinal section of the nodule showed by pre-MWA ultrasound. MWA = microwave ablation.

The patient was a 44-year-old female patient who had presented with thyroid nodules for at least 5 years, had a history of atrial premature beats, and who had undergone an ovariohysterectomy almost 10 years previously. There was no history of hypertension, diabetes, or other infectious disease and allergies, except for hepatitis B. Thyroid ultrasound suggested a 15 × 35 mm solid cystic nodule located in the upper dorsal side of the right lobe of the thyroid gland (Fig. 1). The nodule was well defined with regular form. Streaky bloodstream signals were observed in the interior and edges of the nodule. Pre-MWA thyroid function tests showed a thyroid-stimulating hormone level of 0.912 uIU/mL, free T3 of 4.61 pmol/L, free T4 of 13.30 pmol/L, thyroglobulin antibody of 14.46 IU/mL, and thyroid peroxidase antibody of 37.61 IU/mL.

After being admitted from the outpatient department, the patient completed the remaining pre-MWA examinations. The results of an electrocardiogram, laryngoscopy, and lung computed tomography scan were all normal. The patient was given a principal diagnosis of nodular goiter. We decided to perform MWA given the small volume and benign character of the nodule. We used a MWA instrument (ECO-100A1; YIGAO Microwave System Engineering Co. Ltd, Nanjing, China), matched aseptic disposable MWA needle (ECO-100AL3; 100 mm in length, 1.6 mm in diameter), and 500 mL normal saline for cold fluid circulation for the ablation procedure. The output power setting was 35 W with a frequency of 2450 MHz. Moreover, ultrasound (GE, LogiQ-E9) was used for guidance before, during, and after the ablation.

The patient underwent MWA in November 2017. Considering that local anesthesia would not adequately reduce pain, talking, or coughing during the MWA procedure, we injected lidocaine into the skin puncture site with the assistance of intravenous anesthesia. The patient subsequently fell asleep and therefore, we did not monitor ptosis during the procedure. After confirming the effect of the anesthesia, we set up a liquid-isolating zone by injecting 10 mL normal saline into the space between the anterior capsule of the thyroid gland and the cervical anterior muscles, between the lateral capsule of the thyroid gland and the carotid artery, between the posterior capsule of the thyroid gland and the recurrent laryngeal nerve crossing area, and between the esophagus and the parathyroid gland. Next, we performed MWA from the deep to the shallow part of thyroid gland. The procedure was completed successfully.

**Figure 2:**
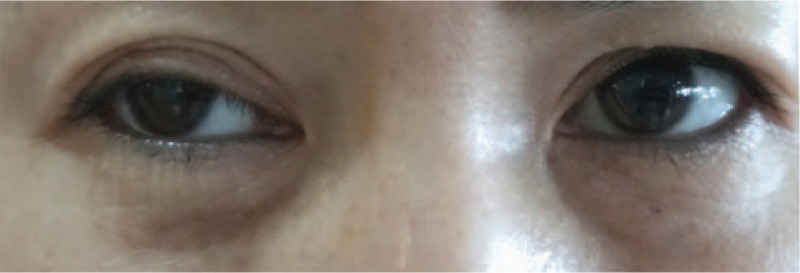
The patient showed miosis and eyelid ptosis in her right eye after 1 month of MWA. MWA = microwave ablation.

Subsequently, we advised the patient to remain laying down and abstain from drinking water for at least 2 hours. About 4 hours after MWA, the patient showed mild miosis and eyelid ptosis in her right eye but no enophthalmos, anhidrosis, or vascular dilatation. Her symptoms became more serious a day later and therefore, the patient underwent brain magnetic resonance imaging and examination by a neurological physician. Along with the clinical presentation, these assessments ruled out the possibility of oncothlipsis and the patient was finally diagnosed with HS as a rare complication of MWA (Fig. 2). Therefore, routine treatment with mecobalamin was administered immediately.

**Figure 3:**
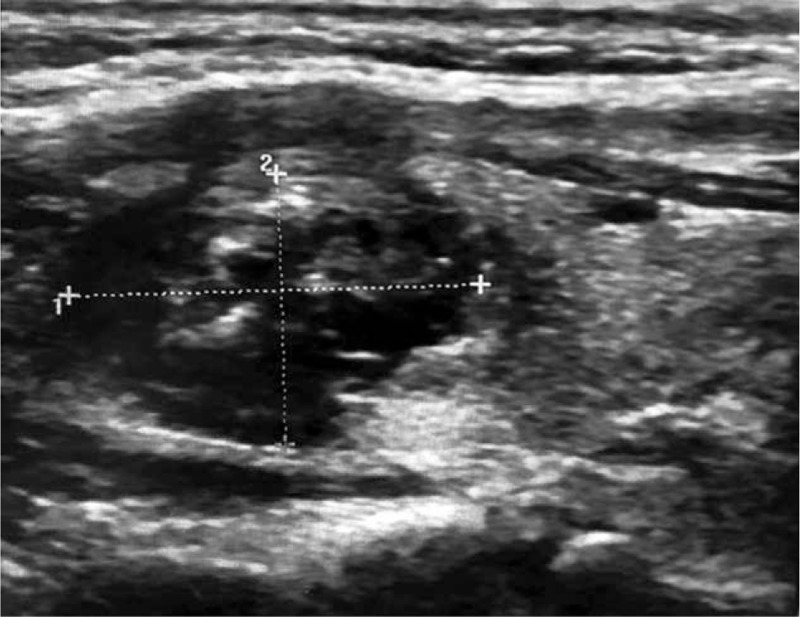
Longitudinal section of the nodule showed by ultrasound after 42 days of MWA. MWA = microwave ablation.

After 42 days of MWA, the patient's nodule showed a reduction in volume with ultrasound (Fig. 3). However, after 5 months of follow up, the patient's miosis and ptosis had not been completely alleviated.

## 3 Discussion

There have been a few reported cases of HS after thyroid surgery, including thyroidectomy, thyroidectomy with lateral lymph node dissection, and most commonly, video-assisted thyroidectomy. However, HS after MWA has not yet been reported in the literature. MWA is a method of percutaneous thermal ablation, similar to RFA and with an equally reliable curative effect.^[7,8]^ However, MWA uses higher energy and is mainly used for larger thyroid nodules. It is suggested that RFA should be adopted for smaller nodules (≤2 cm) or by beginners because it has less power and a smaller volume of damage per unit time, allowing the user to adjust the energy according to the patient's reaction at any time. In contrast, experienced operators often recommend MWA because of its shorter ablation time and because it is relatively less painful.

In our department, the use of MWA to treat benign thyroid nodules (especially those with a diameter under 3 cm) has been observed to have several benefits, including being safe, efficient, and cost effective. Moreover, it is minimally invasive, leaving a good cosmetic appearance without scars. It is less traumatic than the alternatives, with a rapid recovery and no need for extra levothyroxine sodium intake. However, every surgical procedure is associated with risks and even such a minor procedure is not an exception. The complications of MWA are similar to those of traditional surgery: recurrent laryngeal and superior laryngeal nerve injury, post-MWA hypocalcemia, surgical area hemorrhage, etc. Despite these risks, we suggest that MWA is still uniquely superior to other treatments and its benefits should not be ignored.

HS is caused by any compression or destruction of the oculosympathetic pathway (OSP), and it can lead to miosis (with normal reaction to light), eyelid ptosis, enophthalmos, ipsilateral facial anhidrosis, and vascular dilatation.^[9]^ It can be classified into 3 types, central, preganglionic, and postganglionic, depending on the damaged region.^[10]^ The cervical sympathetic trunk is located among the common carotid artery, the internal carotid artery, the vagus nerve, and the anterior fascia of the vertebra, with superior, intermediate, and inferior cervical ganglia. The sympathetic nerve is also distributed throughout the iris sphincter muscle, dilatator iridis, glandula lacrimalis, and cerebrovascular system. Once stimulated, it can cause corectasis, increased secretion of the lacrimal gland, and cerebral vasoconstriction.

Cervical sympathetic nerve syndrome occurs when conduction of the sympathetic nerve is blocked. Generally, the cervical sympathetic trunk will not be damaged if the posterior medial side of the carotid sheath is not involved during a cervical lateral operation, and so it is necessary to pay attention to this region. However, OSP injury is the main cause of the condition with pathological changes occurring following interruption of the OSP, which is associated with cervical lateral trauma surgery, tumors, and inflammation.

Several explanations for HS have been posited by different people. First, the cervical sympathetic trunk is usually located posteromedially to the carotid sheath, anterior to the longus muscles, and beneath the prevertebral fascia.^[11]^ The cervical sympathetic trunk can pass via the posterior wall of the carotid sheath. Nevertheless, sympathetic anatomical variations may occasionally occur due to individual differences. Therefore, an unexpected injury could occur in some people and cause HS during MWA. Second, Reeve study demonstrated that nerve branches exist between the recurrent laryngeal nerve and the sympathetic trunk in some individuals.^[12]^ Unavoidable damage may possibly happen when isolating and identifying the recurrent laryngeal nerve. Therefore, inappropriate operative manipulation can add extra harm to the sympathetic nerve under such circumstances. Third, a vein reported by Solomon originating from the inferior thyroid artery may supply the sympathetic trunk.^[13]^ Ischemia of the sympathetic chain can occur while ligaturing this vessel, which leads to HS. Fourth, post-MWA hematoma and inflammation can be another reason causing dysfunction of sympathetic nerve via compression.^[14]^ Fifth, the vagus nerve, which is surrounded by the carotid sheath and adjacent to the common carotid artery, the internal carotid artery, and the internal jugular vein, must be explored during the operation. However, a single stretching of the carotid sheath or sympathetic nerve can increase the risk of damage. Sixth, the surgical field of vision is too small to present all branches of the vessels and nerves under minimally invasive video-assisted thyroidectomy, especially in ultrasound-guided MWA in which it is virtually impossible to present the sympathetic nerve. Finally, with the popularity of energy-based surgical instruments, cutting, and coagulation with a unipolar electroknife, bipolar electrocoagulation forceps, an ultrasonic scalpel, and an MWA needle provides easier ways for us to perform such procedures. Indeed, it is the protein coagulation effect produced by heat energy instruments that make the MWA procedure faster and more effective. Nonetheless, the sympathetic nerve is also sensitive to heat exposure and this is precisely what causes injury to the nerve. Similarly, Carlander^[15,16]^ reported a high rate of transient recurrent laryngeal never palsy associated with generous use of ultrasonically-activated instruments.

From the above discussion, we can reasonably conclude that there were likely overlapping reasons for HS in our patient who underwent MWA. A common factor among conditions resulting from oculosympathetic damage is the anatomical variation between individuals that can also lead to disruption of important vascular and nerve branches. Another contributing factor is the compression of post-MWA hematoma and inflammation. Perhaps the primary factor was the poor ability to see during surgery under the guidance of ultrasound. However, the fundamental cause is the high heat generated by the active tip of the electrode in MWA, which creates neural lesions.

As equipment and techniques develop, thyroid MWA provides a promising treatment modality. It is generally well received by patients, who report cosmetic satisfaction and positive experiences. Although HS is a rare complication of thyroid MWA, surgeons should be aware of the anatomic relationship of the cervical sympathetic trunk and thyroid gland with adjacent structures. Here, our case report highlights the possible mechanisms of HS, as reported in the literature. We hope by reviewing and reporting these, surgeons are able to take precautionary measures to minimize the possibility of damage.

## Author contributions

**Investigation**: Xi Zhang, Peiyou Ren, Jia Liu.

**Supervision**: Jia Liu, Guang Chen.

**Writing** – **original draft**: Xi Zhang, Yunhao Ge.

**Writing – review & editing**: Xi Zhang, Yunhao Ge.

**Conceptualization:** Xi Zhang.

**Investigation:** Xi Zhang, Peiyou Ren, Jia Liu.

**Resources:** Xi Zhang.

**Supervision:** Jia Liu, Guang Chen.

**Validation:** Xi Zhang.

**Writing – original draft:** Xi Zhang, Yunhao Ge.

**Writing – review & editing:** Xi Zhang, Yunhao Ge.
